# Rhinovirus infection induces cytotoxicity and delays wound healing in bronchial epithelial cells

**DOI:** 10.1186/1465-9921-6-114

**Published:** 2005-10-10

**Authors:** Apostolos Bossios, Stelios Psarras, Dimitrios Gourgiotis, Chrysanthi L Skevaki, Andreas G Constantopoulos, Photini Saxoni-Papageorgiou, Nikolaos G Papadopoulos

**Affiliations:** 1 Allergy Department, 2nd Pediatric Clinic, University of Athens, Athens, Greece

## Abstract

**Background:**

Human rhinoviruses (RV), the most common triggers of acute asthma exacerbations, are considered not cytotoxic to the bronchial epithelium. Recent observations, however, have questioned this knowledge. The aim of this study was to evaluate the ability of RV to induce epithelial cytotoxicity and affect epithelial repair in-vitro.

**Methods:**

Monolayers of BEAS-2B bronchial epithelial cells, seeded at different densities were exposed to RV serotypes 1b, 5, 7, 9, 14, 16. Cytotoxicity was assessed chromatometrically. Epithelial monolayers were mechanically wounded, exposed or not to RV and the repopulation of the damaged area was assessed by image analysis. Finally epithelial cell proliferation was assessed by quantitation of proliferating cell nuclear antigen (PCNA) by flow cytometry.

**Results:**

RV1b, RV5, RV7, RV14 and RV16 were able to induce considerable epithelial cytotoxicity, more pronounced in less dense cultures, in a cell-density and dose-dependent manner. RV9 was not cytotoxic. Furthermore, RV infection diminished the self-repair capacity of bronchial epithelial cells and reduced cell proliferation.

**Conclusion:**

RV-induced epithelial cytotoxicity may become considerable in already compromised epithelium, such as in the case of asthma. The RV-induced impairment on epithelial proliferation and self-repair capacity may contribute to the development of airway remodeling.

## Background

The bronchial epithelium plays a unique role as a protective physical and functional barrier between external environment and underlying tissues. As a result of this role it is frequently injured and epithelial integrity is damaged. A repair process starts quickly which includes migration of the remaining basal airway epithelial cells to repopulate damaged areas, and subsequent proliferation and differentiation until epithelial integrity has been restored [1,2].

Epithelial damage is a key feature of asthma. As a result of inflammation, a large portion of columnar epithelial cells shed and form Creola bodies, detected in sputum and during bronchoscopy in asthmatic patients [3]. This cycle of damage and repair has been proposed as a key mechanism leading to thickening of the airway wall, and other pathologic alterations collectively characterized as airway remodeling [4], which in turn has been associated with incompletely reversible airway narrowing, bronchial hyper-responsiveness and asthma symptoms [5].

Many factors can be cytotoxic to the bronchial epithelium, including eosinophil products [6], allergens [7] and respiratory viruses. Virus-induced cytotoxicity has been well documented for the majority of these agents, including influenza, parainfluenza, adenovirus and respiratory syncytial virus (RSV) [8]. In contrast, human rhinoviruses (RVs), although the most preponderant viruses associated with acute asthma exacerbations [9], have been shown to induce minimal, if any, cytotoxicity [10-12]. We have recently shown that RVs are able to replicate in human primary bronchial epithelial cells [13]. An unexpected finding in that study was that exposure of sparely seeded cell monolayers resulted in a considerable RV-specific cytopathic effect (CPE). RV-induced CPE was also reported in another study, in which case it was attributed to specific RV serotypes [14].

Based on the above, we hypothesized that RV infection may be conditionally able to affect epithelial cell viability and life-death cycle. Therefore, in this study we used BEAS-2B cells, a well-established in-vitro lower respiratory epithelium model of RV infection [15,16], used in parallel studies with primary bronchial epithelial cells [17,18]as well in cell death studies [19]to systematically investigate the ability of RV to induce cytotoxicity in bronchial epithelial cells. Furthermore, the effect of RV on an in-vitro model of epithelial wound repair was assessed.

## Methods

### Cell cultures

BEAS-2B cells, a human continuous bronchial epithelial cell line and Ohio-HeLa cells (obtained from ATCC and the MRC Cold Unit, UK, respectively) were cultured in Eagle's minimal essential medium (E-MEM) buffered with NaHCO_3 _and supplemented with 10% (v/v) fetal bovine serum (FBS) and 40 μg/ml of gentamycin, in a humified 5% CO_2 _incubator. Cells were spilt twice weekly.

Primary human bronchial epithelial cells (HBECs), initially deriving from an adult non-asthmatic volunteer in the course of another ongoing study, were available frozen in liquid nitrogen. They were isolated as described earlier [13]. Cells were rapidly thawed and cultured on plates pre-coated with collagen type-I (Nutacon, Holland), submerged in Clonetics BEGM (Cambrex, ML, USA). Medium was replaced daily. Cells were used at passage 2, at a confluence of 50%. It should be pointed out that cells in these submerged cultures are undifferentiated and do not form tight junctions [20].

All culture reagents were purchased from Gibco-Invitrogen Corp. (Carlsbad, CA, USA) and Falcon (Becton Dickinson, Labware, NJ, USA) and biochemicals were from Sigma (St. Louis, MO, USA), unless otherwise specified.

### Virus cultures and titration

Rhinovirus types 5, 7, 9, 14 and 16 (major subtypes) and 1b (minor subtype) were propagated in Ohio-HeLa cells in large quantities at 33°C, in a humified, 5% CO_2 _incubator, as previously described[16]. Briefly, when full cytopathic effect (CPE) developed, cells and supernatants were harvested, pooled, frozen and thawed twice, clarified, sterile-filtered, aliquoted and stored at -70°C. Lysates of parallel Ohio-HeLa cell cultures, not infected with virus, were used as controls.

In order to determine RV titers, Ohio-HeLa cells were seeded in 96-well plates reaching 60–70% confluence at the time of infection. Logarithmic dilutions of RVs were made in multiple wells and after five days of culture the plates were fixed and stained with Crystal Violet Buffer (5% formaldehyde, 5% ethanol and 0.1% crystal Violet in PBS). The end-point titer was defined as the highest dilution at which a CPE was detected in at least half of the wells and expressed as the inverse logarithm of this dilution (MOI-multiplicity of infection-infectious units/cell). For each experiment, a new vial was rapidly thawed and used immediately [21].

In order to assess the specificity of RV-mediated responses, RV preparations were exposed to 58°C for 1 h. The successful inactivation was confirmed by lack of RV replication in Ohio-HeLa cells.

### Cytotoxicity assay

BEAS-2B cells were plated in 48-well plates in serial dilutions and allowed to grow for 48 hours, reaching confluence of 100%, 50%, 25% and 12.5%. Cell numbers and respective confluence were assessed by standard Neubauer cytometer in initial experiments. Cells were then exposed to rhinovirus as previously described [16]. Briefly wells were washed with HBSS and virus was added at the desirable MOI in parallel to non-infected Ohio HeLa cell lysate negative controls. The amount of the virus added was proportional to the number of the cells. After 1 hour of gentle shaking at room temperature, fresh medium was added, to a final volume of 0.5 ml. Eagle's MEM supplemented with 4% FCS, 1% MgCl and 4% tryptose phosphate broth and 40 μg/ml of gentamycin, was used for the experiments. After 48 hours of incubation, cells were washed twice in PBS and a volume of crystal violet staining buffer equal to 1/5^th ^of the original culture medium was added to the wells as indicator of cell viability [22,23]. Cells were incubated for 30 min at room temperature followed by extensive washing with distilled water. After air drying 0.2 ml of a destain buffer (16.6% v/v glacial acetic acid, 50% v/v methanol in ultra pure water) was added to the wells for 5 min. Cells were fully destained and the produced color was transferred to a clear 96 well ELISA plate and optical density was measured with a photometer at 595 nm (Ceres 900C, Bio-Tec Instruments, Inc, Winooski VT, USA) [13]. Cytotoxicity was estimated as % of the negative control (1- O.D RV infected/O.D HeLa * 100).

### Epithelial repair assay

Confluent monolayers of BEAS-2B cells were grown in 48-well plates. Cells were then damaged mechanically by crossing three times with a 10–200 μL volume universal pipette tip (Corning, NY, USA) [1]. After washing twice with HBSS cells were infected with RV1b at MOI 0.5, in parallel to non-damaged monolayers as well as HeLa lysate controls as described above and incubated in a humified 5% CO_2 _incubator. Immediately after infection (t = 0) and at 24, 48 and 72 hours a plate was stained with crystal violet. Wells were photographed and the area of unpopulated cells was calculated with image analysis, (Scion Image software, b4.0.2, NIH). Furthermore, cytotoxicity was estimated, as described above.

In some experiments, cells were fixed and stained with 4, 6 diamino-2-phenylindole (DAPI), a DNA-binding dye. They were then viewed using a UV-visible Zeiss Axioplan 2 fluorescent microscope and fluorescence images were captured using a CCD camera.

### Proliferation Assay

BEAS-2B cells were cultured in 25 cm^2 ^flasks until confluent. After infection with RV 1b at 0.5 MOI or control, cells were incubated for an additional 24 hours at 33°C. They were then washed twice with HBSS, detached using a non-enzymatic cell dissociation buffer (Gibco, UK), split 1:2 in new flasks and re-incubated. At that time, as well as at 24, 48 and 72 hours later proliferation was estimated by staining with Proliferating Cell Nuclear Antigen (PCNA), a proliferation marker correlates with other markers of the S phase of cell cycle like tritiated thymidine and Bromodeoxyuridine labeling [24]. PCNA assessed with flow cytometry [25].

### Flow Cytometry

BEAS-2B cells were harvested non-enzymatically and resuspended at a density of 1 × 10^5 ^cells/100 μl in washing buffer (PBS with 1% FBS). For ICAM-1 analysis cells were incubated with 20 μL anti-ICAM, phycoerythin-conjugated monoclonal antibody (Pharmingen, Becton Dickinson, Jan Hose, CA, USA) for 30 min at 4°C. After washing twice, cells were fixed with 0.5 ml of 1% paraformaldehyde in PBS and counted with a FACSort (Becton Dickinson, Jan Hose, CA, USA) flow cytometer. Fluorescence data were collected on 10^4^cells and histogram analysis was performed with the use of Cell Quest software™.

For PCNA analysis, cells were permeabilized in a buffer comprising of 0.2 mg/ml Na_2_HPO_4_-2H_2_O, 1 mg/ml KH_2_PO_4_, 45% v/v acetone and 9.25% v/v formaldehyde [25], followed immediately by staining with 10 μL of an anti-PCNA, fluorescein-conjugated monoclonal antibody (Pharmingen, Becton Dickinson, Jan Hose, Ca, USA). Fluorescence data from 10^4^ cells were collected and histogram analysis was performed with Cell Quest software.

Cell viability was assessed by staining with 7-aminoactinomycin D (7-AAD) (Becton-Dickinson, San Jose, Calif., USA).

### Statistical Analysis

Data are expressed as mean ± standard error of mean. Statistical analysis was conducted with the SPSS 11.0 for Windows software. Linear regression analysis was used to evaluate the effect of cell density, and ANOVA for time and dose comparisons. Means were compared by non-parametric tests. P values less than 5% were considered significant.

## Results

### Rhinoviruses induce cytotoxicity in bronchial epithelial cells in a serotype and cell density- depended manner

**Figure 1:**
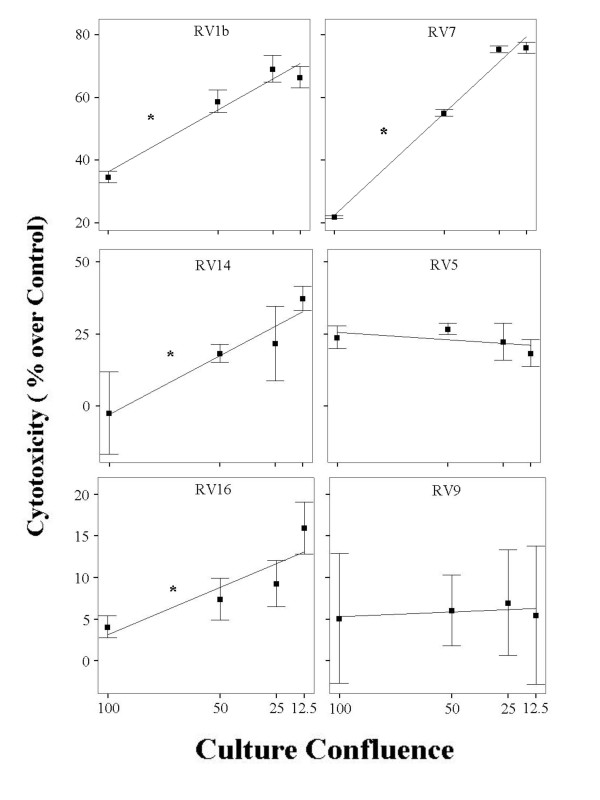
Cytotoxicity of different RV serotypes; 1b, 7, 14, 5, 16 and 9, at MOI = 1 on BEAS-2B cells, cultured until reaching different densities (100, 50, 25 and 12.5%). An inverse correlation between cell density and RV-induced cytotoxicity is observed for RV1b, RV7, RV14 and RV16, (*p < 0.05, n = 3–26, linear regression).

BEAS-2B cultures were infected with RV1b, RV5, RV7, RV9, RV14 and RV16 at an MOI of 1 and confluences of 12.5%, 25%, 50% and 100%. The extent of RV-induced cytotoxicity differed between RV serotypes: RV9 was not cytotoxic at all at this MOI. RV1b and RV7 were the most cytotoxic, able to induce cytotoxicity even on confluent monolayers, while killing over 65%–70% of less dense cultures. RV14 and RV5 were moderately cytotoxic while RV16 could kill only sparsely seeded cells. Differences in RV-induced cell death between RV serotypes were statistically significant at all cell densities (p = 0.00 in all cases, ANOVA). Furthermore, a statistically significant inverse correlation between cell density and RV-induced cytotoxicity was observed for RV1b, RV7, RV14 and RV16, (p = 0.000, 0.000, 0.014 and 0.03 respectively, linear regression); RV5 was moderately cytotoxic at all cell densities. Figure 1 shows the % cytotoxicity of each RV serotype at different cell densities.

HBEC infected with 1 MOI of RV 1b, RV7 and RV16 at 50% confluence (n = 4), showed cytotoxicity levels of 50 ± 1%, 52 ± 3% and 0% respectively, almost identical to those observed in BEAS-2B cells under the same conditions, confirming that the described phenomenon is reproducible in primary cells.

### Rhinovirus-induced cytotoxicity is dose depended

**Figure 2:**
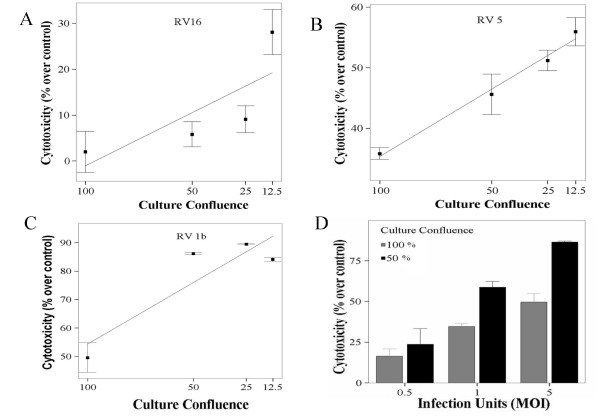
Cytotoxicity of RV16 (A), RV5 (B) and RV1b (C) at MOI = 5 on BEAS-2B cells, cultured until reaching different densities (100, 50, 25 and 12.5%, n = 3–8). RVs became more cytotoxic at this MOI, and density dependence appeared for RV5. Dose dependence is shown for RV1b (D) at 0.5, 1, and 5 MOI for both 100% and 50% cell densities (p = 0.000 in both cases, n = 6, ANOVA).

Subsequently, dose-dependence of cytotoxicity was assessed using RV1b, RV5, RV9 and RV16. At MOI-5, RV9 remained non-cytotoxic (data not shown). RV16 (Figure 2A), but even more RV 5 (Figure 2B), became cytotoxic on a cell-density dependent manner (p = 0.007 and p = 0.000, respectively, linear regression). RV1b at an MOI of 5 was able to kill almost 50% of a confluent monolayer, reaching a plateau of 85%–89% of cytoxicity in less dense cultures (Figure 2C). A dose-response was observed when comparing cytotoxicity of RV1b both in 100% confluent (16.5% ± 4.45, 34.66% ± 1.85, 49.65% ± 5.24 at 0.5, 1 and 5 MOI respectively, p = 0.000, ANOVA) and 50% confluent monolayers (23.76% ± 9.66, 58.75% ± 3.62, 86.46% ± 0.62 at 0.5, 1 and 5 MOI respectively, p = 0.000, ANOVA) (Figure 2D).

### RV- induced cytotoxicity is specific

**Figure 3:**
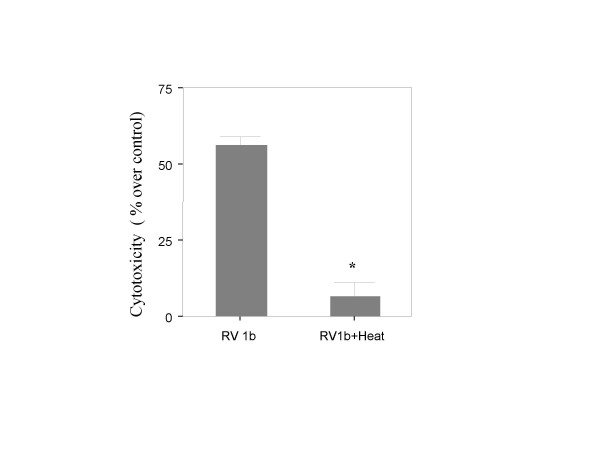
Cytotoxicity of active and heat inactivated RV1b (MOI = 1) on 50% confluent BEAS-2B cells. Inactivated virus is no longer cytotoxic (*p = 0.021, n = 4, Mann Whitney).

To determine whether the observed cytotoxicity is specific to RV and not associated with factors in the virus preparation, we exposed a 50% confluent monolayer to 1 MOI of heat-inactivated RV 1b. Inactivated RV1b lost its capacity to induce cell death (6.43% ± 3.68 vs. 55.87% ± 2.68 of live virus, p = 0.021, Mann-Whitney) (Figure 3).

### ICAM-1 expression is not affected by cell density

To test whether cell-density dependent, differential susceptibility of BEAS-2B cells to RV cytotoxicity may relate to variations of ICAM-1 expression, the major RV receptor, cells were cultured as described above and expression of ICAM-1 was measured by flow cytometry. In all densities cells expressed ICAM-1 over 98.5%, without differences on fluorescence intensity (1030.6 ± 88.76, 980 ± 48.47, 964.29 ± 25.52 and 987.38 ± 37.5, at cell densities of 100%, 50%, 25% and 12.5% respectively).

### RV infection delays epithelial wound repair

**Figure 4:**
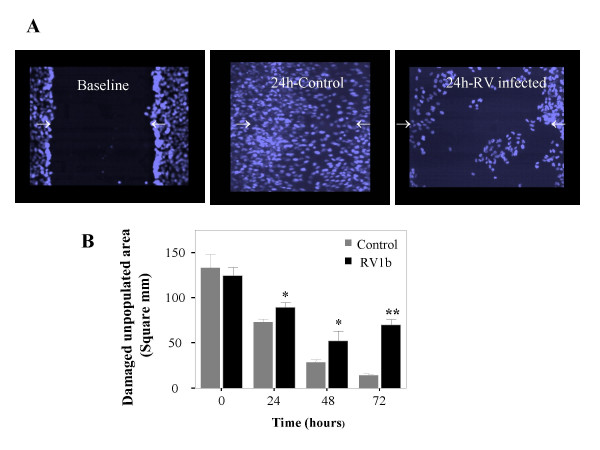
Damaged epithelium (t = 0) is suboptimally repopulated after RV-infection in comparison to control BEAS-2B cells. DAPI stained cells (A). Repopulation of damaged epithelium, expressed as unpopulated area in mm^2^, in RV-infected and non-infected BEAS-2B cells, immediately after damage (t = 0) and at 24, 48 and 72 hours later (B). Repopulation in infected cells is significantly reduced (*p < 0.05, **p = 0.001, n = 8, Mann Whitney).

To test whether infection with RV may affect the self-repair capacity of bronchial epithelial cell monolayers, digital photos were taken immediately after mechanical damage (t = 0) as well as 24, 48 and 72 hours later in infected and non-infected monolayers. The damaged area not populated with cells was calculated by image analysis. Control cells demonstrated a fast response in repopulating the damaged area (from 133.07 ± 14.67 mm^2 ^at t = 0 to 72.92 ± 3.59, 28.09 ± 3.11 and 13.49 ± 1.9 at 24, 48 and 72 hours respectively, p = 0.000, ANOVA). The damaged area in infected monolayers was repopulated considerably more slowly, while it seemed to plateau at 48 hours (t = 0, 124.41 ± 9.26 mm^2^, t = 24 h, 89.13 ± 5.55, t = 48 h, 52.18 ± 10.5, t = 72 h, 69.5 ± 6.3, p = 0.00, ANOVA). When infected and non-infected cells were compared, differences were significant at all time points (p = 0.024, 0.031 and 0.001 at 24, 48 and 72 hours respectively, Mann Whitney), (Figure 4).

Furthermore, intact and wounded monolayers did not differ in susceptibility to RV-mediated cytotoxicity (17.13% ± 4.11 versus 17.64% ± 2.6 at 48 hours after infection for intact and wounded respectively), suggesting that epithelial wounding leaves unaffected the remaining cells of the monolayer in respect to RV-induced cytotoxicity.

### RV infection decreases epithelial cell proliferation

**Figure 5:**
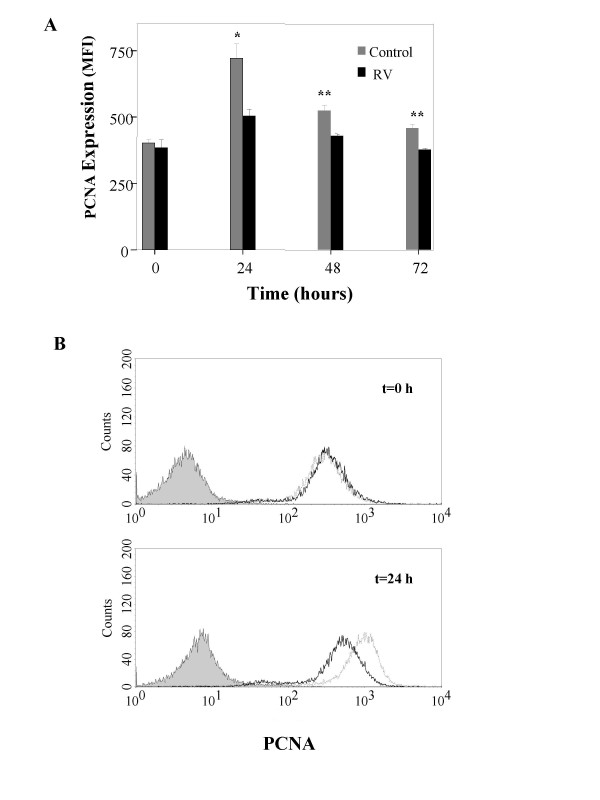
Proliferation of BEAS-2B cells, as mean fluorescence intensity (MFI) of PCNA in RV-infected and control cells at various time points after reculture (A). The proliferation rate is significantly reduced for RV-infected cells at all time points (*p = 0.012, ** p < 0.05, n = 3–7, Mann Whitney). Representative histograms at t = 0 and 24 hours are shown (B). Closed line: isotype control, thick line: RV-infected, dashed line: non-infected control cells.

The expression of PCNA, reflecting proliferative activity of epithelial cells, increased 24 hours after seeding, followed by a trend towards return to baseline at 48 and 72 h. However, PCNA expression (Mean Fluorescence Intensity, MFI) was significantly lower at all time points in RV-infected cells (Figure 5). Cell viability, assessed by 7ADD staining, was over 90% in these experiments.

## Discussion

In contrast to previous knowledge, but in line with recent observations, this study demonstrates that human rhinoviruses, the agents most frequently associated with acute asthma exacerbations [26], are able to become cytotoxic in an in-vitro model of human bronchial epithelium. A continuous cell line model was used for the majority of experiments; however, the finding was also confirmed in primary bronchial cells. Furthermore, it is shown for the first time that RV infection may delay epithelial wound healing by affecting epithelial cell proliferation.

It has been generally accepted that RVs do not induce cytotoxicity in-vitro or in-vivo [27-29], even in heavy colds [10-12,30]. However, two recent studies designed to assess the ability of RV to infect primary human bronchial epithelial cells have unexpectedly observed RV-associated cytotoxicity: in the study of Schroth et al [14], RV16 and RV49 were used and cytotoxicity was observed only with the latter serotype; the authors hypothesized that a higher viral binding and/or larger yield by RV49 may explain their observation, noting however the need for additional studies. This was also the case in the study of Papadopoulos et al. [13] in which RV cytotoxicity was observed when sparsely seeded cultures were exposed to the virus. The current study, which systematically addressed these possibilities, demonstrates that they are both reproducible, and in fact different RV serotypes differ in their cytotoxic capacity, which in most cases, is nevertheless cell density dependent. The latter finding can also explain why RV cytotoxicity was not observed in previous in-vitro studies, which were conducted with confluent cultures [28,29].

A recent comprehensive study from Deszcz et al [31] is in support of our findings, as it shows that RV14 can induce high levels of cytotoxicity in a bronchial epithelial cell line 16HBE14o^-^. Furthermore, they demonstrate that a possible mechanism is the induction of apoptosis via the mitochondrial pathway, a phenomenon also shown in primary cells from asthmatic subjects [32].

It has been shown that differentiated bronchial epithelial cells grown in air-liquid interface and developing tight junctions, are considerably resistant to RV infection [20]. In this respect, the results of this study, using submerged cultures that lead to non-differentiated cells, may overestimate the in-vivo situation. However, a characteristic of asthma is the significant loss of columnar epithelial cells leading to loss of its integrity and density [4], epithelial damage also correlates to the severity of the disease [33] In this respect, sparsely seeded bronchial epithelial cell cultures, can be considered as an extreme, but relevant model of asthmatic epithelium. Under such conditions, as show herein, RV-associated cytotoxicity increases considerably, with almost linear density-dependence, suggesting that virus-induced exacerbations may have increased sequels in more severe patients [34].

The fact that different RV serotypes are not equally capable of killing epithelial cells, ranging from no to extensive cytotoxicity, supports the possibility that this phenomenon may contribute to asthma exacerbation severity variations observed in clinical practice [35].

RV infects a small proportion of exposed cells [15]; biopsy data show that in human RV infection epithelial inflammation, potentially resulting from infection, is patchy [27,36]: there has been, however, no direct comparison between normal and asthmatic individuals in regard to RV-induced cytotoxicity in-vivo, a study complicated by the fact that the epithelial integrity and viability is considerably affected in asthmatics at baseline. In a recent study, Wark et al showed increased RV proliferation in primary epithelial cells obtained from asthmatic patients in comparison to normal controls [32]. We have also observed that exposure of BEAS-2B cells to culture supernatants modeling an 'atopic' environment, was also able to increase RV proliferation, and at the same time increase RV-induced cytotoxicity [37]. These observations further suggest that RV-induced cytotoxicity may be relevant in asthma exacerbation pathogenesis.

There are several possibilities in respect to the mechanism(s) underlying this phenomenon, which have not, however, been addressed in this study. One possibility might be that rapidly dividing cells, as is the case of sparse cultures, may be more permissive to RV infection. Moreover, differential expression of soluble factors, such as interferons, may regulate either susceptibility to infection or the proliferative potential of RV. These hypotheses, which may well not be mutually exclusive and could all contribute to RV cytotoxicity, are currently under investigation.

Independent of the causative mechanism(s), in an already affected epithelium, RV infection may lead to more profound damage. This would eventually lead to activation of repair mechanisms: deposition of extracellular matrix, proliferation and migration of epithelial cells in order to repopulate the damaged area, followed by cell differentiation [1,2]. Hence, we used a previously validated wound model [38,39] to investigate the role of RV infection on the repair process, describing for the first time an RV-mediated delay in epithelial wound healing, associated with reduced proliferation of RV infected cells. This finding may be of significance as altered restitution of airway structure is one of hallmarks of asthmatic inflammation leading to airway remodeling [4]. Damaged asthmatic epithelium has been previously reported to have proliferation defects during the repair process [40]. Dysregulated proliferation in bronchial epithelial cells from asthmatic patients has been associated with increased expression of the cyclin-dependent kinase inhibitor p21waf [41,42]. In severe, corticosteroid-dependent asthma, markers of epithelial cell proliferation are coexpressed with markers of activation, suggesting that, in at least that case, the repair process is associated with a persistent activation state of the epithelial cells [41]. The above findings have led investigators to propose that a repair/activation imbalance may be the central mechanism of airway remodeling in asthma [5]. In this respect, RV-induced cytotoxicity, an event frequently occurring and able to activate epithelial cells into an inflammatory response [16], may be implicated in the development of remodeling. The possibility that a viral infection may reprogram epithelial responses towards a 'remodeled' phenotype has also been proposed, based on a mouse model of paramyxoviral infection [43].

## Conclusion

In conclusion, several human RV serotypes are able to become cytotoxic to human bronchial epithelial cells, especially when these are sparsely cultured; RVs are also able to delay epithelial wound healing. Previously unrecognised, RV-induced cytotoxicity may become important in the context of asthma in which the epithelium is already affected and consequently contribute to the induction and/or perpetuation of airway remodelling.

## Competing interests

The author(s) declare that they have no competing interests.

## Authors' contributions

AB carried out the major part of experiments, participated in the sequence alignment and drafted the manuscript.

SP participated in epithelial repair assay.

DG participated in the study design and in the sequence alignment.

CLS performed primary epithelial cell experiments

PSP participated in the study desigh and helped to draft the manuscript.

AGC participated in the sequence alignment.

NGP, conceived of the study, participated in its design and coordination and participated in the writing of the manuscript.
